# Dental Materia Medica

**Published:** 1866-03

**Authors:** Geo. Watt


Vol. XX.] MARCH 1866. [No. 3.

### Original Communications.

### DENTAL MATERIA MEDICA.

BY GEO. WATT.

### Persulphate of Iron,—$Fe_2O_3, 3SO_3$.

Iron forms several compounds with oxygen. When these elements are united in the proportions of one equivalent of iron and one and a half of oxygen, or, which is the same thing, two of iron and three of oxygen, the compound is called sesquioxyd of iron. With this oxyd, three equivalents of sulphuric acid unite to form the salt under consideration; hence it is a tersulphate of the sesquioxyd of iron.

Preparation.—This salt may be prepared by several processes. It is usually obtained by acting on the protosulphate of iron, thus:

Dissolve a definite quantity of the protosulphate in pure water; add to the solution half as much sulphuric acid as the protosulphate contains; boil the mixture, and while boiling, add to it successive portions of nitric acid as long as orange-colored, or red fumes are given off; then evaporate to dryness. The remaining salt is the *persulphate of iron.*

It will not often be necessary for the dentist to prepare it for his own use, as most energetic druggists keep it on hand.

This, however, was not the case a few years ago. As its solution is often more convenient for the dentist than the powder, he need not always evaporate to dryness, in preparing it, but may stop when the liquid has gained the consistence of a thin syrup.

Physiological Effects.—Persulphate of iron acts on living tissues, by virtue of its affinity for albumen, with which it combines, forming a pale yellowish compound. The salt appears to unite directly with albumen, without undergoing any decomposition; and the compound, thus formed, is much harder and firmer than the clot resulting from the coagulation of fibrin, and is not soluble in an excess of either of its ingredients.

Administered internally, it acts as a general astringent, and is regarded, by many, as a valuable medicine in the treatment of hemorrhagic diathesis.

Uses.—The dentist needs it only to arrest hemorrhage resulting from extraction, or from lancing the gums. As its local application, assisted by pressure, will almost invariably arrest the bleeding, and as there are other internal remedies better, or at least better known, for such purposes, he is much more concerned with its topical than with it constitutional uses. It may be applied in substance or solution, on pledgets of cotton or lint, or on pieces of prepared spunk. As the clot which it forms with albumen is quite brittle, it is very important, in extreme cases, to apply pressure, to hold it in place, thus plugging the mouths of the bleeding vessels, till the fibrin within them coagulates and adheres to their walls, and so permanently closes them.

This salt is a most reliable styptic. A styptic, to be worthy of the name, must form, with some constituent of the blood, a solid compound not soluble in blood; and, to be of practical value in the mouth, the clot must be insoluble in saliva. The persulphate fulfills both these requirements to the highest degree. Many have learned to rely on it in practice, without a correct knowledge of its *modus operandi.*

I have promptly and permanently closed with it arteries as large as medium-sized timothy straws, which had been exposed and divided by the removal of tumors. Twice I have obliterated aneurisms of the palatal artery, by laying them open by free incisions, and forcing into them pledgets of lint, saturated with a concentrated solution of it, and applying pressure by means of a plate previously fitted to the roof of the mouth.

### Perchloride of Iron.—$Fe_2Cl_3$.

This salt, though but a sesquichloride, represents, as its name imports, the highest degree of chloridation of which iron is known to be capable.

Preparation.—The perchloride of iron is readily obtained by dissolving sesquioxyd of iron (jewelers' rouge) in pure hydrochloric acid, and evaporating the solution to dryness. Or if it is wanted in solution, evaporate only to a syrupy consistence. Or it may be conveniently and successfully prepared by dissolving iron in *aqua regia*, composed of two or three parts of hydrochloric, and one part of nitric acid, and evaporating as above. The reactions which take place may be represented by the following equation:

$$Fe_2 + 3HCl + NO = Fe_2Cl_3 + 3HO + NO_2.$$

Phisiological Effects and Uses. — These are so nearly similar to those of the persulphate just considered, that but little need be said in this connection. The perchloride forms, with albumen, a softer and much less brittle compound than the persulphate. It is, therefore, more likely to succeed without the aid of pressure; but in all obstinate cases, pressure should not be neglected — neither omitted nor carelessly applied.

### Tannin, or Tannic Acid.—$C_{18}\ H_8\ O_{12}$.

Most of the vegetable astringents owe their activity to the presence of this agent. It is found abundantly in oak bark and nutgalls. The latter contain 35 or 40 per cent. of it.

It is prepared by percolation, or "displacement," with ether. But as it is kept in all drugstores, the dentist will never be called on to prepare it for his own use.

PROPERTIES.—Without being bitter, tannin has an intensely astringent taste; as found in commerce, its color is a lemon yellow; and it is often beautifully crystallized. With a solution of gelatin (glue) it forms a white precipitate, *tannate of gelatin*. With solutions of persalts of iron, it forms *tannate of iron*, a bluish-black compound, used extensively as writing ink.

PHYSIOLOGICAL EFFECTS.—Tannic acid is an active astringent. It has a powerful affinity for albumen, fibrin, etc., its medicinal effects depending on this principle. Its powers are lessened, and its effects localized, by the character of the compound it forms with albuminous tissues Tannate of albumen is not soluble in water, or in any constituent of the blood. Owing to this fact, tannin is a very reliable styptic, more so than any others, except the two just considered. It forms, with the blood, a firmer clot than either of the others; but it is so thin, that, without support from judicious pressure, the blood will burst through it, in severe cases.

USES.—Tannin is used by the dentist to arrest hemorrhage, to allay sensibility of dentine, to cure chaped lips, and to counteract a spongy condition of the gums. For the latter purpose, the tincture of galls, or a solution of tannin in diluted alcohol, will afford the best preparation. On sensitive dentine, its action is so superficial, that it does not accomplish much. The first portion of it that comes in contact with the gelatinous part of the tooth, forms with it an insoluble layer, which shuts out subsequent portions. Hence, little or nothing is gained by repeated applications of it. Its solution in glycerine and water is an admirable application to sore lips. The dentist often finds it necessary to cure the lips, in order to operate, especially on the posterior teeth. Glycerine and rosewater, each half an ounce, and tannin ten grains, mixed, applied three or four times a day, will answer a good purpose.

As a styptic, tannin may be relied on, with the aid of pressure, to the exclusion of all other substances, except the persulphate and perchloride of iron. The mode of applying it, and the means of pressure, will suggest themselves to the dentist.

